# *Helicobacter pylori* CagA Induces Cortactin Y-470 Phosphorylation-Dependent Gastric Epithelial Cell Scattering via Abl, Vav2 and Rac1 Activation

**DOI:** 10.3390/cancers13164241

**Published:** 2021-08-23

**Authors:** Nicole Tegtmeyer, Aileen Harrer, Klemens Rottner, Steffen Backert

**Affiliations:** 1 Department of Biology, Division of Microbiology, Friedrich-Alexander University Erlangen-Nuremberg, Staudtstr. 5, 91058 Erlangen, Germany; Aileen.Harrer@anatomie.med.uni-giessen.de (A.H.); steffen.backert@fau.de (S.B.); 2 Department of Cell Biology, Helmholtz Centre for Infection Research, 38124 Braunschweig, Germany; kro@helmholtz-hzi.de; 3 Division of Molecular Cell Biology, Zoological Institute, Technische Universität Braunschweig, 38106 Braunschweig, Germany

**Keywords:** *Helicobacter*, cancer, cortactin, pathogenesis, pathogenicity island, signaling, virulence

## Abstract

**Simple Summary:**

Various microbial pathogens target the actin-binding protein cortactin to promote their own uptake, proliferation and spread, and exhibit proposed roles in human cancerogenesis. We aimed to study the molecular mechanisms of how the gastric pathogen *Helicobacter pylori* hijacks cortactin phosphorylation via tyrosine kinase Abl to trigger cancer-related signal transduction events. We discovered that cortactin phosphorylated at Y-470 recruits the signaling factor Vav2 to activate the small Rho GTPase Rac1, and finally, a cancer cell motility phenotype. We also demonstrate that phosphorylation of cortactin at Y-470 can be completely inhibited by the well-known Abl inhibitor imatinib. Imatinib is an established oral chemotherapy medication, employed for efficient systemic treatment of various cancers. These results reveal a comprehensive novel pathway for how precisely *H. pylori* manipulates host signaling in gastric disease development, and may pave the way for new opportunities of treatment of the outcome of infections with this pathogen, i.e., through using imatinib.

**Abstract:**

The pathogen *Helicobacter pylori* is the first reported bacterial type-1 carcinogen playing a role in the development of human malignancies, including gastric adenocarcinoma. Cancer cell motility is an important process in this scenario, however, the molecular mechanisms are still not fully understood. Here, we demonstrate that *H. pylori* subverts the actin-binding protein cortactin through its type-IV secretion system and injected oncoprotein CagA, e.g., by inducing tyrosine phosphorylation of cortactin at Y-470, which triggers gastric epithelial cell scattering and motility. During infection of AGS cells, cortactin was discovered to undergo tyrosine dephosphorylation at residues Y-421 and Y-486, which is mediated through inactivation of Src kinase. However, *H. pylori* also profoundly activates tyrosine kinase Abl, which simultaneously phosphorylates cortactin at Y-470. Phosphorylated cortactin interacts with the SH2-domain of Vav2, a guanine nucleotide exchange factor for the Rho-family of GTPases. The cortactin/Vav2 complex then stimulates a previously unrecognized activation cascade including the small GTPase Rac1, to effect actin rearrangements and cell scattering. We hypothesize that injected CagA targets cortactin to locally open the gastric epithelium in order to get access to certain nutrients. This may disturb the cellular barrier functions, likely contributing to the induction of cell motility, which is important in gastric cancer development.

## 1. Introduction

Infection by *H. pylori* is the strongest known risk factor for the development of various gastric diseases, including stomach cancer [1,2]. The latter disease represents an important cancer type in humans, which accounted for approximately one million new cases in the year 2020 with about 769,000 deaths, ranking at position four for mortality and five for incidence worldwide [3]. *H. pylori* deregulates inflammation, cell proliferation, scattering, and motility [1,2]. These activities support *H. pylori* survival in the host, but also initiate gastric diseases. Cell migration and involved actin rearrangements are fundamental activities in various cellular processes in healthy cells, but also in cancer cell progression [4,5,6]. The multifaceted accomplishments during cell migration, invasion and metastasis are regulated by dynamic processes in the cortical actin structures, employing an array of signaling proteins that stimulate specific rearrangements in the architecture of the cytoskeleton [7,8]. Among them are important signal transduction molecules, such as small GTP-hydrolyzing proteins (GTPases) of the Rho family that are switched into the active states by a set of guanine nucleotide exchange factors (GEFs). Accordingly, several microbial pathogens developed strategies in evolution to hijack the host cytoskeletal apparatus during infection to promote their uptake, proliferation and spread [9,10,11]. One of these successful pathogens is *H. pylori*, a major risk factor for gastric diseases and malignancies [1,2,3]. *H. pylori* takes control over various signal transduction cascades in the host, regulating processes like cell proliferation, scattering, motility and inflammation [1,2]. These activities support *H. pylori* survival in the host, but also initiate gastric diseases. Highly virulent *H. pylori* isolates utilize the cag type IV secretion system (T4SS) to deliver the effector protein CagA into the gastric epithelium. Afterwards, injected CagA is phosphorylated initially by Src and subsequently Abl tyrosine kinases [12,13], which is associated with cytoskeletal rearrangements resulting in cell scattering and elongation [14,15,16,17]. The small GTPase Rac1 is also activated and may play a role in these responses, however, the exact activation pathway and potentially involved GEFs have so far remained unknown [18,19]. Moreover, injected CagA can bind to multiple signaling factors, one of which is the kinase Csk, which phosphorylates Src at Y-527, negatively regulating its catalytic activity [20,21]. Src inactivation by this negative feedback-loop mechanism results in profound tyrosine dephosphorylation of Src substrates, including cortactin [21]. Cortactin appears to be a major target of *H. pylori*, however, the individual tyrosine residues in cortactin and their importance in signaling have not yet been investigated.

Cortactin is a multi-domain and multi-faceted actin binding protein, known as a central integrator of signal transmission to actin cytoskeleton remodeling [22]. Cortactin had originally been described as a Src kinase substrate and was implicated in cell migration and invasion [23], as well as tumor metastasis [7]. Cortactin comprises an N-terminal acidic (NTA) domain that encodes a typical DDW-sequence, described to bind and/or activate the Arp2/3 complex [7,24]. An array of filamentous actin (F-actin) binding domains and a proline-rich linker follows. This linker region includes various PxxP-motifs and multiple phosphorylation sites. For example, cortactin can be phosphorylated at Y-421, Y-470 and Y-486 by the tyrosine kinases Src, Syk, Fer and Abl, and at S-405 and S-418 by the serine/threonine kinases PAK1and ERK [22]. Finally, a Src homology 3 (SH3) domain forms the C-terminal end of cortactin. This SH3-domain permits the interaction with multiple binding partners such as N-WASP, WIP, MLCK, FAK, dynamin-2 and others [7,22,25,26,27,28]. Notably, the binding of N-WASP promotes Arp2/3-mediated actin-polymerization independent of the NTA domain of cortactin in vitro [29], which stimulates N-WASP recruitment to actin nucleation sites in invadopodia and podosomes in vivo [30] and appeared to enhance cellular motility [31]. In the present study, we aimed to investigate in detail the role of tyrosine phosphorylation and dephosphorylation events of cortactin at tyrosines 421, 470 and 486 by *H. pylori* CagA, and of related signal transduction pathways leading to scattering and motility of infected gastric epithelial cells.

## 2. Materials and Methods

### 2.1. Cell Culture, Bacteria and Inhibitors

Human AGS gastric adenocarcinoma cells (CRL-1739) were purchased from ATCC (Manassas, VA, USA). The cells were incubated in RPMI-1640 medium with 10% FCS at 37 °C and 5% CO_2_ [32]. The Abl kinase inhibitors (2.5 µM imatinib and 1 µM SKI-DV-43) were added 30 min prior to infections, according to the manufacturer and as listed in Table S1. The *H. pylori* wild-type strains P1 and P12, as well as the corresponding isogenic Δ*cagA* deletion mutants were cultivated on GC agar plates in anaerobic jars with CampyGen gas packs [33]. The bacteria were resuspended in BHI medium using sterile cotton swabs. The OD_600_ nm was measured by an Eppendorf spectrophotometer, and *H. pylori* were added to cells at a multiplicity of infection (MOI) of 50. Infection times are given in each figure legend.

### 2.2. Cell Fractionation, Immunoprecipitation and Western Blotting

After infections, AGS cells were collected in ice-cold PBS supplemented with 1 mmol/L Na_3_VO_4_ (Sigma-Aldrich, St. Louis, MO, USA). Biochemical fractionation of infected cells as cytosolic and membrane fractions was performed as described previously [28]. Immunoprecipitation and Western blotting were performed according to published standard protocols [34,35]. Band intensities on Western blots were quantified using the Image Lab software (Bio-Rad, Hercules, CA, USA). The strongest signal on each blot was set as 100%. All antibodies are listed in Table S1, and were handled according to the respective manufacturers’ protocols, as listed. Full Western Blots can be found in File S1.

### 2.3. Plasmids, Mutagenesis and Transfection of DNA and siRNA

Murine cortactin cDNA (1641 bp) was cloned into pEGFP-C1 vector (Clontech, Saint-Germain-en-Laye, France) using *Bgl*II and *Kpn*I restriction sites. The Y466F, Y466D, Y421/466/482D and Y421/466/482F cortactin mutant constructs were generated using QuikChange mutagenesis kit (Stratagene, Santa Clara, CA, USA). EGFP-fusion proteins of Vav2 wild-type, Vav2 Y172/159F, Vav2 R425C, Vav2 W673R and Vav2 G693R variants cloned into EGFPC2 vector (Clontech, Saint-Germain-en-Laye, France) were kindly provided by L. Buday (Research Centre for Natural Sciences, Budapest, Hungary) [36]. HA-tagged human Vav2 was kindly provided by S. Moores (Harvard Medical School, Boston, MA, USA). Transfection of all plasmid constructs into AGS cells was performed using TurboFect transfection reagent following manufacturer’s instructions (Thermo Fisher Scientific, Waltham, MA, USA). After 48 h, transfected AGS cells were infected with *H. pylori*. Successful transfections were checked by Western blotting. For siRNA knock-down studies in AGS cells, cortactin (# sc-35093, Santa Cruz Biotechnology, Dallas, TX, USA), Vav2 (# sc-41738, Santa Cruz Biotechnology, Dallas, TX, USA), Rac1 (# sc-36351, Santa Cruz Biotechnology, Dallas, TX, USA) and scrambled control siRNA oligonucleotides (# sc-37007, Santa Cruz) were transfected for 48 h as described by the supplier (Santa Cruz Biotechnology, Dallas, TX, USA, Table S1), followed by *H. pylori* infection [37].

### 2.4. Wound Healing Assay

AGS cells were cultivated to produce confluent monolayers in 6-well tissue culture plates. A pipette tip was then used to generate a wound. Cell debris was removed by two washing steps using fresh RPMI-1640 medium. Afterwards, cells were infected with *H. pylori* wt for 24 h or 48 h, respectively. Wound closure was examined by phase contrast microscopy DMI4000B (Leica, Wetzlar, Germany). To quantify cell migration, changes in wound areas were measured over time. The number of elongated AGS cells was determined in parallel, as described [28,38].

### 2.5. Rac1 GTPase Activity Assay

The activation status of Rac1 in the presence or absence of *H. pylori* was determined with a Rac1 Activation Kit, using the supplier’s protocol (Cytoskeleton, Denver, CO, USA). For this purpose, AGS cells were grown in 6-well plates for two days, followed by serum-starvation for 12 h. Infected and uninfected cells were then washed with ice-cold PBS buffer, followed by addition of lysis buffer and cell harvesting. A centrifuge step (for 5 min at 8000× *g*) followed, and total protein concentrations were quantified by Bradford assay (Bio-Rad, Hercules, CA, USA). Two milligrams of total proteins were applied in every assay. The resulting cell lysates were then mixed with PAK-RBD slurry, followed by incubation at 4 °C on a shaker for 1 h at 4 °C [39,40]. Afterwards, the beads were centrifuged for 3 min at 4000× *g*, followed by two washing steps in washing buffer. The pellets were then re-suspended in 50 µL 1× SDS buffer and subjected to Western blotting.

### 2.6. Live Cell Imaging

For live cell imaging studies, serum-starved AGS cells cultivated on 12-well plates were infected with *H. pylori* wt for 6 h in the presence or absence of the Abl inhibitor imatinib, and monitored using the JuLI™ Smart fluorescent cell analyzer (NanoEnTek Inc., Waltham, MA, USA) placed in a CO_2_ incubator at 37 °C.

### 2.7. Statistical Analysis

All experiments were done at least in triplicates. All data were evaluated using one-way ANOVA followed by Tukey’s test with GraphPad Prism statistical software (version 8.0). Statistical significance was defined by *p* ≤ 0.05 (*), *p* ≤ 0.01 (**) and *p* ≤ 0.001 (***).

## 3. Results

### 3.1. Subcellular Localization of Phosphotyrosine-Cortactin after Infection with H. pylori

**Figure 1:**
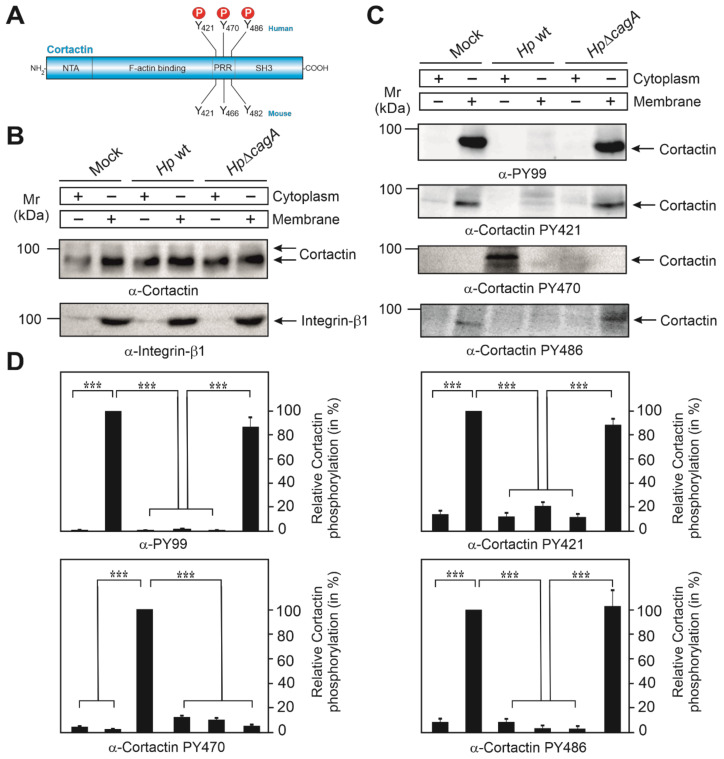
Cortactin undergoes dephosphorylation at tyrosine residues Y-421 and Y-486, and phosphorylation at Y-470 during *H. pylori* infection. (**A**) Schematic representation of the cortactin domain structure. Three tyrosine phosphorylation sites in human cortactin are shown on top, their mouse counterparts on the bottom. (**B**) AGS cells were co-incubated with wt *H. pylori* or Δ*cagA* mutant for 6 h. Mock control and infected cells were harvested and separated into cytoplasmic and membrane fractions. The loading control blots for cortactin and integrin-β_1_ are shown. (**C**) Phosphorylation of cortactin at tyrosine residues 421, 470 and 486 was investigated with the indicated phosphotyrosine-specific antibodies. (**D**) Densitometric quantification of cortactin tyrosine phosphorylation bands of Western blots in panel C. Error bars represent +/− standard deviation (SD). The results are from three independent experiments. *p*-values of ≤0.001 (***) were considered as statistically significant.

We have previously shown that *H. pylori* targets cortactin upon infection of gastric epithelial cells [21,28]. Here, we investigate the role of tyrosine-phosphorylated cortactin during infection of AGS gastric epithelial cells. Human cortactin harbors three tyrosines phosphorylated by Src and other kinases, Y-421, Y-470 and Y-486, corresponding to tyrosines 421, 466 and 482 in murine cortactin, respectively (Figure 1A). We previously used the pan-phosphotyrosine antibody PY-99, which strongly recognizes tyrosine-phosphorylated cortactin [21,28]. However, this antibody does not discriminate between individual phosphorylation sites in cortactin. To overcome this, we utilized commercial phospho-specific antibodies directed against tyrosines 421, 470 and 486. To this end, AGS cells were infected with wild-type (wt) *H. pylori* or Δ*cagA* mutant for 6 h, followed by biochemical fractionation into cytoplasmic and membrane fractions. As proper loading controls, staining with a non-phospho α-cortactin antibody revealed similarly strong signals in every fraction, while the basolateral integrin-β_1_ receptor revealed only bands in the membrane, but not in the cytosol fraction, as expected (Figure 1B). These samples were probed with all available phosphotyrosine-specific α-cortactin antibodies. In agreement with downregulation of Src activity in our previous reports [12,21,28], the α-PY-99 antibody recognized tyrosine-phosphorylated cortactin in the membrane fraction of uninfected mock control cells and Δ*cagA* mutant infected cells, but not in the wt infected samples (Figure 1C, top panel). This confirmed that cortactin specifically undergoes tyrosine-dephosphorylation upon wt *H. pylori* infection in a CagA-dependent manner. A similar pattern was observed when using α-PY421 and α-PY486 antibodies. In contrast, the PY470-specific antibody revealed a band only in the cytosol fraction of wt-infected AGS cells. This suggested that the α-PY-99 antibody only recognizes cortactin phosphorylated at Y-421 and Y-486, but not Y-470. In addition, these results demonstrated that cortactin was not completely tyrosine-dephosphorylated, but only at two sites (Y-421 and Y-486), while cortactin phosphorylated at Y-470 was specifically induced by *H. pylori* in a CagA-dependent fashion. Quantitations are shown in Figure 1D.

### 3.2. Cortactin Y-470 Is Phosphorylated by Activated Abl Kinase, Correlating with Cell Scattering 

**Figure 2:**
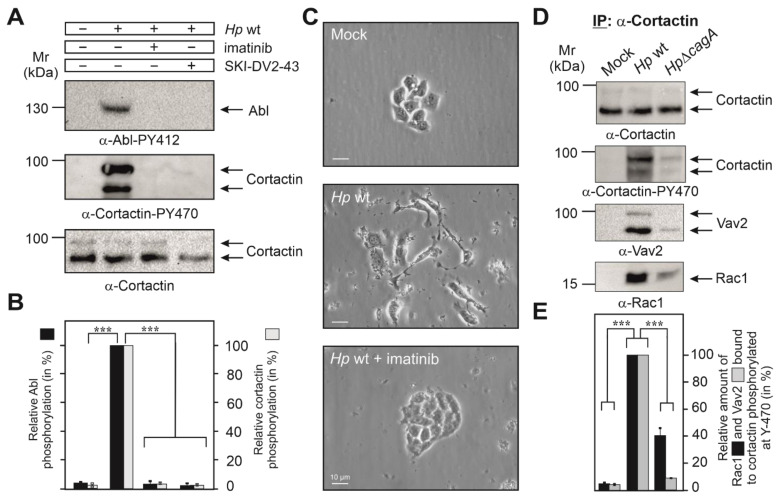
Cortactin Y-470 is phosphorylated by activated Abl kinase and forms a physical complex with Vav2 and Rac1, associated with cell motility. (**A**) AGS cells were infected for 6 h with *H. pylori* in the presence or absence of the Abl-specific inhibitors imatinib and SKI-DV2-43. Cell lysates were probed with indicated antibodies, demonstrating the activated Abl phosphorylated cortactin at Y-470 (arrows). (**B**) Densitometric quantification of phospho-band intensities for Abl (Y-412) and cortactin (Y-470). (**C**) Phase contrast microscopy revealed that *H. pylori* induces cell scattering, which is abolished by the Abl inhibitor imatinib. 400× magnification, scale bars represent 10 µm (**D**) AGS cells were co-incubated with wt *H. pylori* or Δ*cagA* mutant for 6 h, followed by immunoprecipitation (IP) using cortactin antibodies. Resulting blots were probed with antibodies as indicated. The results show that cortactin phosphorylated at Y-470 interacts with Vav2 and Rac1 in a CagA-dependent manner. (**E**) Band intensities of Rac1 and Vav2 bound to phosphorylated cortactin were quantified using the Image Lab software version 6.1. Error bars represent +/− SD. The results are from three independent experiments. *p*-values of ≤0.001 (***) were considered as statistically significant.

We and others have reported previously that *H. pylori* infection induced the constitutive phosphorylation of Abl kinase at Y-412 in its active center [12,13,14,15]. We thus proposed that activated Abl could phosphorylate cortactin at Y-470. To investigate this possibility, we infected AGS cells for 6 h with wt *H. pylori* in the presence or absence of the Abl-specific inhibitors imatinib or SKI-DV2-43. Resulting blots showed that either inhibitor significantly downregulated both the activity of Abl and phosphorylation of cortactin at Y-470 (Figure 2A,B). In addition, Abl activation and cortactin Y-470-phosphorylation temporally correlated with the induction of AGS cell scattering by *H. pylori* infection, which was also inhibited by imatinib or SKI-DV2-43 (Figure 2C, Videos S1–S3 and [41]). Together, this suggested that Abl indeed phosphorylates cortactin at Y-470, and this correlates with the induction of cell scattering.

### 3.3. Cortactin PY-470 Recruits the GEF Vav2 and Small GTPase Rac1 during Infection

**Figure 3:**
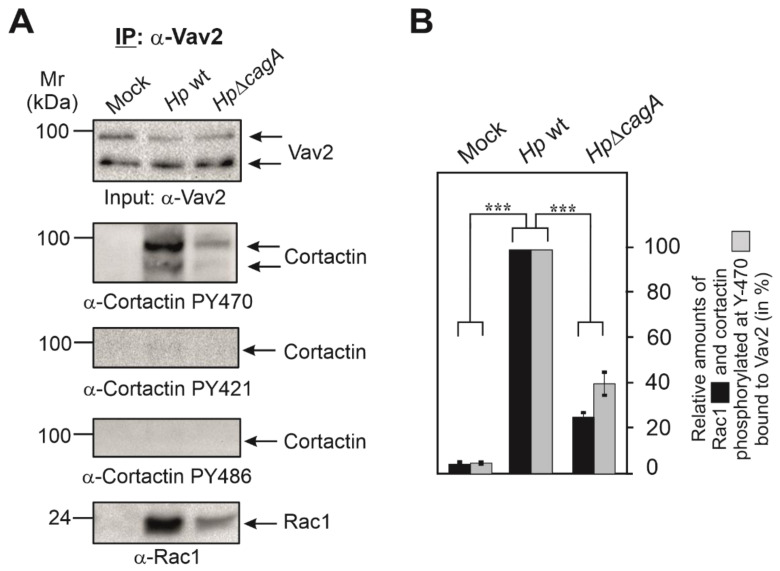
Vav2 forms a physical complex with Rac1 and cortactin phosphorylated at Y-470. (**A**) AGS cells were infected for 6 h with wt *H. pylori* or Δ*cagA* mutant, followed by immunoprecipitation (IP) using Vav2 antibodies. The corresponding Western blots were probed with antibodies as indicated. The results suggest that Vav2 interacts with Rac1 and cortactin phosphorylated at Y-470 in a CagA-dependent fashion. (**B**) Band intensities of Rac1 and phosphorylated cortactin bound to precipitated Vav2 were quantified using the Image Lab Software (Bio-Rad). Error bars represent +/− SD. *p*-value of ≤0.001 (***) was considered as statistically significant.

Assuming that cortactin Y-470 phosphorylation may operate directly in AGS cell scattering led us to hypothesize that a specific GEF might be recruited to cortactin-PY470. To investigate this idea, we infected AGS cells for 6 h with wt or Δ*cagA* mutant *H. pylori*, followed by immunoprecipitation (IP) of cortactin and analysis by Western blotting. Staining with non-phospho α-cortactin antibody revealed similarly strong bands in each lane, confirming cortactin loading at comparable amounts (Figure 2D). However, probing of the samples with α-Cortactin-PY470 antibody revealed a strong band only for the wt-infected sample. These blots were then reprobed with specific antibodies against different, well-known GEFs including Dock180, Tiam, PIXα or Vav2 [40,42]. Remarkably, we only received a strong band for Vav2 in the complex (Figure 2D), but not for the other GEFs [41]. This suggested that PY470-cortactin might bind and perhaps activate Vav2. Since Rac1 activation has been already described for *H. pylori* infection [18,19], we then also probed the IPs with antibodies against Rac1, and could show that Rac1 is present in the same immune-complex of cortactin-PY470 with Vav2 (Figure 2D,E). To provide further evidence for this assumption, we performed the reverse IP experiment using α-Vav2 antibodies. The results verified that Rac1 formed a complex with cortactin-PY470, but not with cortactin-PY421 or cortactin-PY486 (Figure 3A,B). These findings are in line with the hypothesis that cortactin stimulates AGS cell scattering through activating a Vav2-Rac1 signaling axis.

### 3.4. Phosphorylation of Cortactin at Y-470 Is Associated with Increased Rac1-GTP Levels

**Figure 4:**
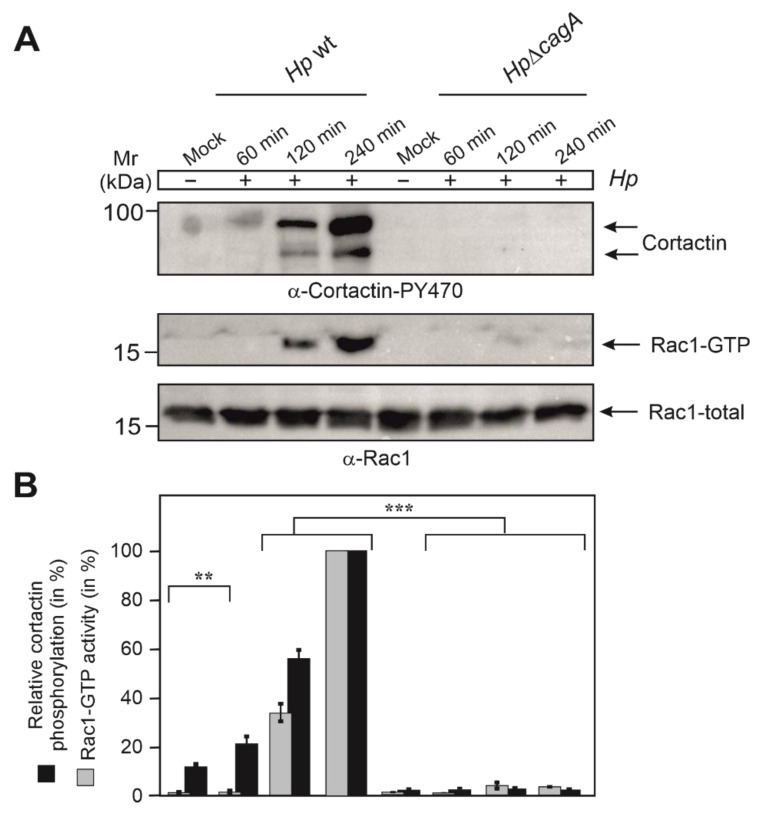
Cortactin phosphorylation at Y-470 temporally correlating with Rac1 activation during *H. pylori* infection. (**A**) AGS cells were infected with *H. pylori* wt or Δ*cagA* mutant for different times, i.e., 60, 120 and 240 min, followed by probing with α-cortactin-PY-470 (top) or Rac1-GTP pulldown and α-Rac1 Western blot (middle). The α-Rac1 blot of total cell lysates served as loading control (bottom). (**B**) Quantification of band intensities on Western blots showing cortactin levels phosphorylated at Y-470 and relative Rac1-GTP activity. The results are from three independent experiments. *p*-values of ≤0.01 (**) and ≤0.001 (***) were considered as statistically significant. The results confirm that the induction of cortactin phosphorylation at Y-470 and activation of Rac1 display a temporal correlation.

Next, we explored if phosphorylation of cortactin at Y-470 and activation of Rac1 correlate. For this purpose, AGS cells were infected for distinct times with wt or ∆*cagA* mutant *H. pylori*. Resulting cell lysates were subjected to Western blotting and Rac1 activation assays. The results showed that wt *H. pylori,* but not ∆*cagA* mutant, induced the predominant phosphorylation of cortactin at Y-470 between 2 and 4 h of infection (Figure 4A,B). Strikingly, those time points precisely reflected the induction of active GTP-bound Rac1 in those experimental conditions, whereas ∆*cagA* mutant induced Rac1 activation only to minute extents (Figure 4A,B). These results clearly demonstrate the precise temporal correlation between cortactin phosphorylation at Y-470 and Rac1 activation.

### 3.5. Vav2 Binds to Cortactin via Its SH2 Domain

**Figure 5:**
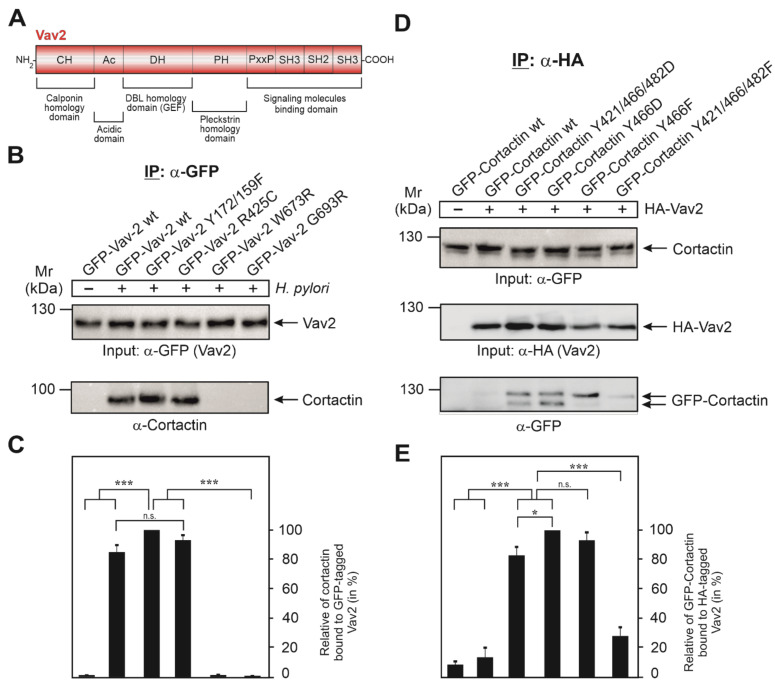
Vav2 binds via its SH2 domain to cortactin phosphorylated at Y-470. (**A**) Schematic representation of the Vav2 domain structure.. (**B**) AGS cells were transfected with GFP-Vav2 wt and various GFP-Vav2 mutant constructs, as indicated, for 48 h, followed by infection with *H. pylori* for 6 h. Cell lysates were harvested and subjected to IP using α-GFP antibodies to precipitate GFP-Vav2. Similarly, presence of each construct in the IPs was confirmed by α-GFP antibody Western blot (top). This blot was then re-probed with α-cortactin antibody. (**C**) Densitometric quantification of cortactin band intensities bound to precipitated GFP-Vav2. (**D**) AGS cells were transfected with GFP-cortactin wt and various GFP-cortactin phospho-mutant constructs, as indicated, for 48 h in the presence or absence of HA-tagged Vav2 plasmid. Total cell lysates were probed with α-GFP antibodies showing similar amounts of GFP-cortactin proteins present in each lane (top). Parallel samples were subjected to IP using α-HA antibodies to precipitate HA-tagged Vav2 (middle). This blot was then re-probed with α-GFP antibodies to detect bound cortactin (bottom). (**E**) Densitometric quantification of cortactin band intensities bound to precipitated HA-Vav2. The results show that cortactin and Vav2 can bind to each other by a typical phosphotyrosine-SH2 domain interaction. Error bars represent +/− standard deviation (SD). The results are from three independent experiments. *p*-values of ≤0.05 (*), and ≤0.001 (***) were considered as statistically significant. n.s.: not significant.

Since tyrosine phosphorylation of cortactin at Y-470 is associated with Vav2 binding, and such interactions are commonly mediated by binding to SH2 domains, we investigated this possibility for the Vav2-cortactin interaction. Interestingly, the Vav2 domain structure reveals the presence of one SH2 domain near the C-terminus (Figure 5A). Therefore, we next evaluated the role of Vav2 domains by mutation. For this purpose, AGS cells were transfected with GFP-Vav2 wt and different GFP-Vav2 mutant plasmids, i.e., Vav2 lacking its tyrosine phosphorylation site (Vav2-Y172/159F), its PIP3 (phosphatidylinositol 3,4,5-trisphosphate) binding motif (Vav2-R425C), or with a mutated SH2 domain (Vav2-W673R and Vav2-G693R), which was previously found to impair Vav2 binding to the EGF receptor [36]. At 48 h after transfection, the cells were infected with wt *H. pylori* for 6 h, followed by an IP using α-GFP antibodies to precipitate GFP-Vav2. Comparable expression of these constructs was confirmed by Western blotting using α-GFP antibodies (Figure 5B, top). Reprobing of the blots with α-cortactin antibodies revealed only a very faint signal for bound cortactin in the absence of *H. pylori* and a strong band in the presence of *H. pylori* (Figure 5B, bottom). Similarly strong cortactin bands were detected in the IPs with transfected Vav2-Y172/159F and Vav2-R425C, suggesting that tyrosine phosphorylation or PIP3 binding by Vav2 play no role in its interaction with cortactin. In contrast, inactivation of the Vav2 SH2 domain by mutation of W673R or G693R prevented cortactin binding completely (Figure 5B, bottom). For quantitations of bound cortactin signals, see Figure 5C. Together, these findings suggest that cortactin and Vav2 can bind each other by a canonical phosphotyrosine-SH2 domain interaction.

### 3.6. Expression of GFP-Cortactin Point Mutants Confirms Its Requirements for Vav2 Binding 

To dissect the significance of cortactin tyrosine phosphorylation residues in the interaction with Vav2, we utilized a collection of GFP-cortactin constructs, including phosphorylation-deficient point mutants (Y > F) and phosphorylation-mimetic mutations (Y > D) (Figure 5D). These plasmids were co-transfected for 48 h into AGS cells together with HA-tagged Vav2 wt construct. Expression of all these constructs was similar, as confirmed by α-GFP and α-HA Western blots (Figure 5D). In parallel samples, sets of transfected cells were subjected directly to IP with α-HA antibodies. As expected, Western blot results revealed a very faint signal for GFP-cortactin wt (unmodified) bound to Vav2 (Figure 5D, bottom). The strongest signals for bound GFP-cortactin, however, were observed for the phosphotyrosine-mimetic Y421/466/482D and Y466D mutants (mouse constructs), whereas there was a weaker band for the single Y466F mutant or a very faint signal for the triple phosphotyrosine-deficient Y421/466/482F mutant (Figure 5D, bottom). These results confirm that phosphorylation of cortactin at residue Y-466 (as well as tyrosines 421 and 482 to some extent) positively regulates the interaction of cortactin with Vav2 when expressed from constructs (Figure 5E), even in the absence of *H. pylori*.

### 3.7. Cortactin and Vav2 Expression Are Required for Maximal Rac1 Activation 

**Figure 6:**
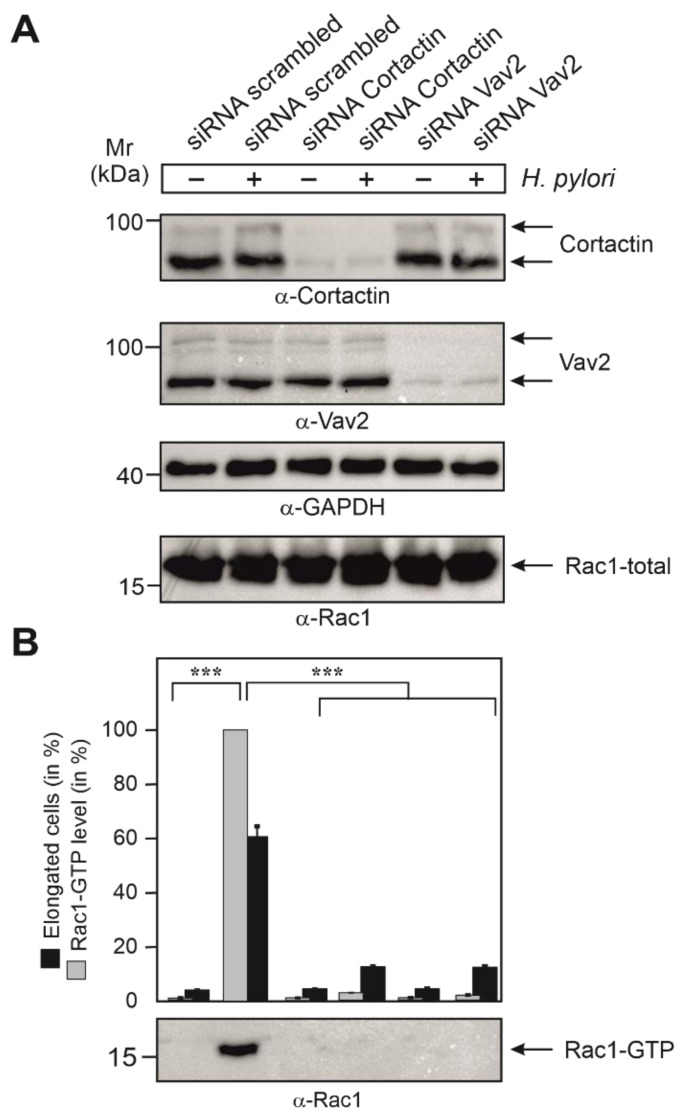
Control blots for the knockdown of cortactin and Vav2 by siRNAs and quantification of Rac1-GTP levels in AGS cells infected with *H. pylori*. (**A**) AGS cells were grown, treated with siRNAs and then infected with *H. pylori* wt, as described in Figure 7. The resulting cell lysates were probed with the indicated antibodies to demonstrate successful downregulation of cortactin and Vav2 expression, respectively, and equal protein loading in each lane. (**B**) A second set of samples, as shown in panel A, were subjected to Rac1-GTP pulldowns. The resulting α-Rac1 blot (bottom) was quantified by densitometric evaluation of the bands employing the luminescence imager. The sample showing the strongest Rac1 signal corresponds to 100% Rac1 activity in the respective blot. Inhibition of Rac1-GTP levels by knockdown of cortactin or Vav2 were quantified in three independent experiments. *p*-values of ≤0.001 (***) were considered to report on statistically significant differences.

As the next step, we aimed to study if the expression of cortactin and Vav2 were required for the activation of Rac1 during infection. For this, we treated AGS cells with siRNAs against cortactin and Vav2, or scrambled siRNAs as control. After 48 h, cells were either infected with wt *H. pylori* or left uninfected. Control blots showed that the expression of cortactin and Vav2 was downregulated in the presence of corresponding siRNAs, but not in scrambled siRNA controls, while the expression of Rac1 and GAPDH were not affected in each sample (Figure 6A). In a parallel experiment, the same set of transfected cells was subjected to Rac1-GTP pulldown assays. Importantly, the strong Rac1-GTP levels induced by wt *H. pylori* were significantly reduced by the downregulation of either cortactin or Vav2 (Figure 6B). These results clearly established that the expression of both cortactin and Vav2 were required for profound activation of Rac1 in *H. pylori*-infected AGS cells.

### 3.8. Expression of Cortactin, Vav2 and Rac1 as Well as Phosphorylation of Cortactin Are Required for Cell Motility Induced by H. pylori 

**Figure 7:**
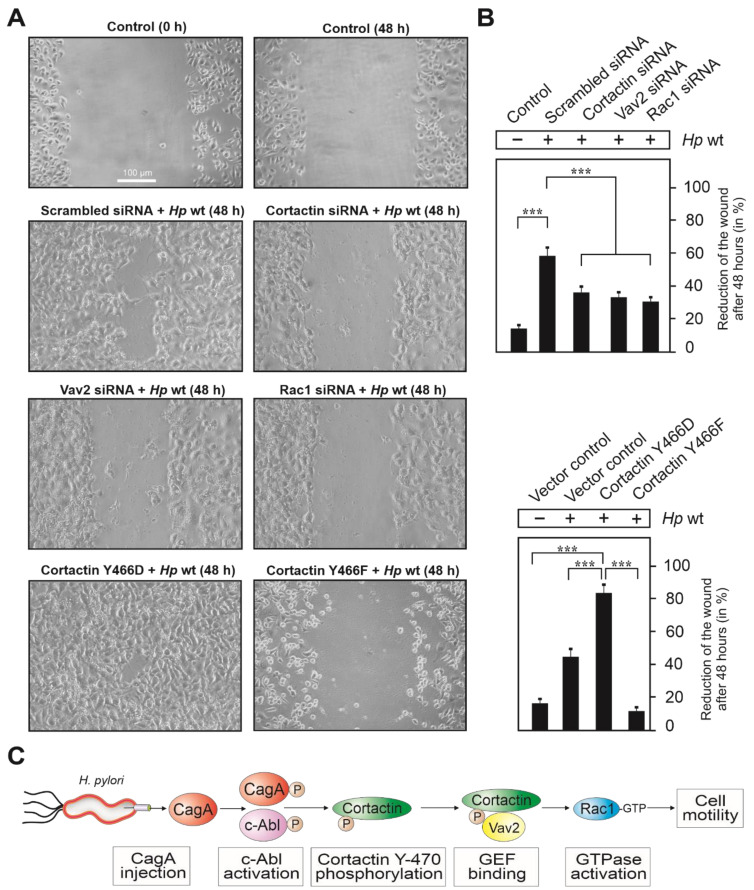
Importance of cortactin, Vav2 and Rac1 expression as well as cortactin tyrosine phosphorylation for AGS cell migration during infection with *H. pylori*. (**A**) Confluent cell monolayers of AGS cells were transfected with siRNAs against cortactin, Vav2 and Rac1, scrambled siRNAs or Cortactin-Y466D, Cortactin-Y466F and vector control constructs. Then, cells were wounded using a pipette tip, followed by infection with *H. pylori* for 48 h (middle and bottom) or treatment with PBS buffer as control (top). All images at 100× magnification, scale bar represents 100 µm. (**B**) The areas following wound closure were measured after 48 h of infection. Error bars represent +/− standard deviation (SD). The results are from three independent experiments. *p*-values of ≤0.001 (***) were considered as statistically highly significant. (**C**) Proposed signal transduction cascade of injected CagA leading successively to activation of Abl kinase, phosphorylation of cortactin at Y-470, Vav2 binding, Rac1 GTPase activation and cell motility.

Finally, we investigated if cell scattering and migration induced by *H. pylori* infection depended on the expression of cortactin, Vav2 and Rac1. To this end, AGS cells were grown to confluency, followed by transfection with siRNAs against cortactin, Vav2 and Rac1, or scrambled siRNAs as control. After 48 h, these cells were subjected to cell migration (wound healing) assays during infection with *H. pylori* (Figure 7). For this purpose, transfected and control AGS cells were scratch-wounded with a pipette tip to generate a gap area, followed by a washing step to remove cell debris and *H. pylori* infection. The wound zone was recorded for 48 h by phase contrast microscopy (Figure 7A). Upon infection with wt *H. pylori* in the presence of scrambled siRNAs, AGS cells revealed strong motility and wound closure compared with non-infected control cells. In contrast, infection of AGS cells transfected with siRNAs, interfering with cortactin, Vav2 or Rac1 expression exhibited strongly reduced wound healing activities. (Figure 7A,B). To investigate the significance of cortactin phosphorylation at Y-470 on wound closure, we also transfected cells with aforementioned cortactin constructs prior to infection. The results show that overexpression of cortactin with phospho-mimetic Y466D (mouse cortactin, equivalent to Y-470 in humans) significantly enhanced wound closure, while the expression of phospho-resistant Y466F had a suppressive effect (Figure 7A,B, bottom). Together, these results confirm that phosphorylation of cortactin at Y-470 positively regulates AGS cell motility and wound healing upon infection with *H. pylori*. The proposed signaling pathway is presented in Figure 7C.

## 4. Discussion

Cell motility is a central feature of countless regular and disease-associated biological processes, such as embryonic growth, anti-microbial defense, wound healing and tumor cell metastasis [4,5,6]. A key function in this scenario is played by small Rho-family GTPases and the Arp2/3 complex, through controlling host actin cytoskeleton remodeling [43,44]. Another important factor is cortactin, which is involved in several actin-associated cellular functions, such as the organization of membrane dynamics and restructuring of actin networks, although the precise functions and molecular regulation of cortactin in these scenarios are not yet entirely clear [22]. Cortactin commonly operates as an adapter protein that interacts with multiple other proteins via its SH3 domain to stimulate various signal transduction cascades [26,28,45].

During *H. pylori* infection of gastric epithelial cells, cortactin was shown to undergo dephosphorylation at tyrosine residues [21] and phosphorylation at serines [28]. While the role of the tyrosine-dephosphorylated form during infection is not yet clear [21], cortactin phosphorylated at S-113 promoted the loss of F-actin binding; and cortactin phosphorylated at S-405 was required for binding and activation of focal adhesion kinase (FAK) [28]. Dephosphorylation of cortactin on tyrosines agree with our previous findings that phosphorylated CagA downregulates Src kinase activity through activating Csk, as demonstrated using the pan-phosphotyrosine antibody PY-99 [12,21,28]. In the present study, we were able to confirm that these tyrosine dephosphorylation events applied to tyrosines 421 and 486 of human cortactin. To our big surprise, however, *H. pylori* induced profound cortactin phosphorylation at Y-470 (Y-466 in mouse), which was mediated by activated Abl tyrosine kinase and not recognized by the pan-PY-99 antibody. Using a series of different molecular methods, including expression constructs, siRNA knockdown, immunoprecipitation, inhibitors, and wound healing motility assays, we could demonstrate that *H. pylori* triggered a Abl→cortactin→Vav2→Rac1 activation cascade to stimulate actin rearrangements and cell scattering (Figure 7C). This pathway appears to be unique and distinct from the previously reported cortactin→Vav2→Rac3 cascade operating in invadopodia formed by breast cancer cells, in which Rac1 was not activated although it was substantially expressed [46].

Cortactin has been also reported as a target of several other microbial pathogens, which change its phosphorylation status for various purposes upon infection. For instance, the extracellular pathogens EPEC (enteropathogenic *E. coli*) and EHEC (enterohemorrhagic *E. coli*) recruit tyrosine-phosphorylated cortactin to actin-rich pedestals, the sites of bacterial attachment [47,48]. In addition, the invasive pathogens *Staphylococcus aureus* and *Neisseria meningitidis* trigger the tyrosine phosphorylation of cortactin during their entry into host target cells [49,50]. Another example is *Shigella flexneri*, which stimulates cortactin tyrosine phosphorylation by activation of Src, followed by binding of the adapter protein Crk to trigger actin polymerization and bacterial cell entry [51,52]. Cortactin also appears to have a role in *Listeria* invasion, but the phosphorylation status is not clear [53,54]. For each of the latter pathogens, host cell entry was described to be associated with activation of Rac1 [55,56,57,58], however, a cortactin-dependent pathway for Rac1 activation has not been described. This might suggest that *H. pylori*, and probably also some other pathogens, utilize the above discovered Rac1 activation pathway via cortactin and Vav2 specifically to induce cell motility (Figure 7C), and not bacterial entry. We propose that *H. pylori* induces the phosphorylation of Y-470 in cortactin to locally open the gastric epithelium at sites of infection, perhaps to retrieve important nutrients likely absent in the gastric lumen, and thus, disturbing cellular barrier functions. This new activity probably works in conjunction with a previously reported function of injected CagA, which is to hijack Par1b kinase for disruption of epithelial cell polarity [59]. In addition, the gene encoding cortactin is amplified in various human cancers [60]. Together with the current findings, this may pinpoint cortactin as a possible biomarker for gastric tumorigenesis.

## 5. Conclusions

Taken together, we demonstrate here that the gastric carcinogen *H. pylori* profoundly induced cortactin phosphorylation at residue Y-470, which triggers cell scattering and elongation with a proposed, accompanying function in gastric tumor cell progression. These results reveal a comprehensive novel pathway via Vav2 and Rac1 signaling for how precisely *H. pylori* manipulates host cell signaling cascades in gastric cancer development. Interestingly, phosphorylation of cortactin at Y-470 through injected CagA can be completely inhibited by the well-known Abl inhibitor imatinib (Figure 2). In fact, imatinib is an efficient systemic treatment that is currently routinely applied in irresectable or metastasized gastrointestinal stromal tumors with about 80% of patients responding to imatinib therapy either with partial remission or stable disease. This underlines the significant impact of our findings in understanding cancer treatment regimes, and may pave the way for new opportunities of treatment of the outcome of infections with *H. pylori*, i.e., through using imatinib.

## Data Availability

The data presented in this study are available on request from the corresponding author.

## Supplementary Materials

The following are available online at https://www.mdpi.com/article/10.3390/cancers13164241/s1, Table S1: Key resources of the present study, Video S1: AGS cells were infected for 6 h with *H. pylori* in the absence of the Abl-specific inhibitor imatinib, Video S2: AGS cells were infected for 6 h with *H. pylori* in the presence of the Abl-specific inhibitor imatinib, Video S3: Mock uninfected control of AGS cells for 6 h, File S1: Whole Western Blots.
