# Supplementary material for: Helicobacter pylori CagA Induces Cortactin Y-470 Phosphorylation-Dependent Gastric Epithelial Cell Scattering via Abl, Vav2 and Rac1 Activation
Source: Cancers (Basel). 2021 Aug 23;13(16):4241. doi: 10.3390/cancers13164241 (PMC8391897; doi:10.3390/cancers13164241)
Supplement: Supplementary file 1 [file cancers-13-04241-s001.zip › File S1.pdf]

Figure 1

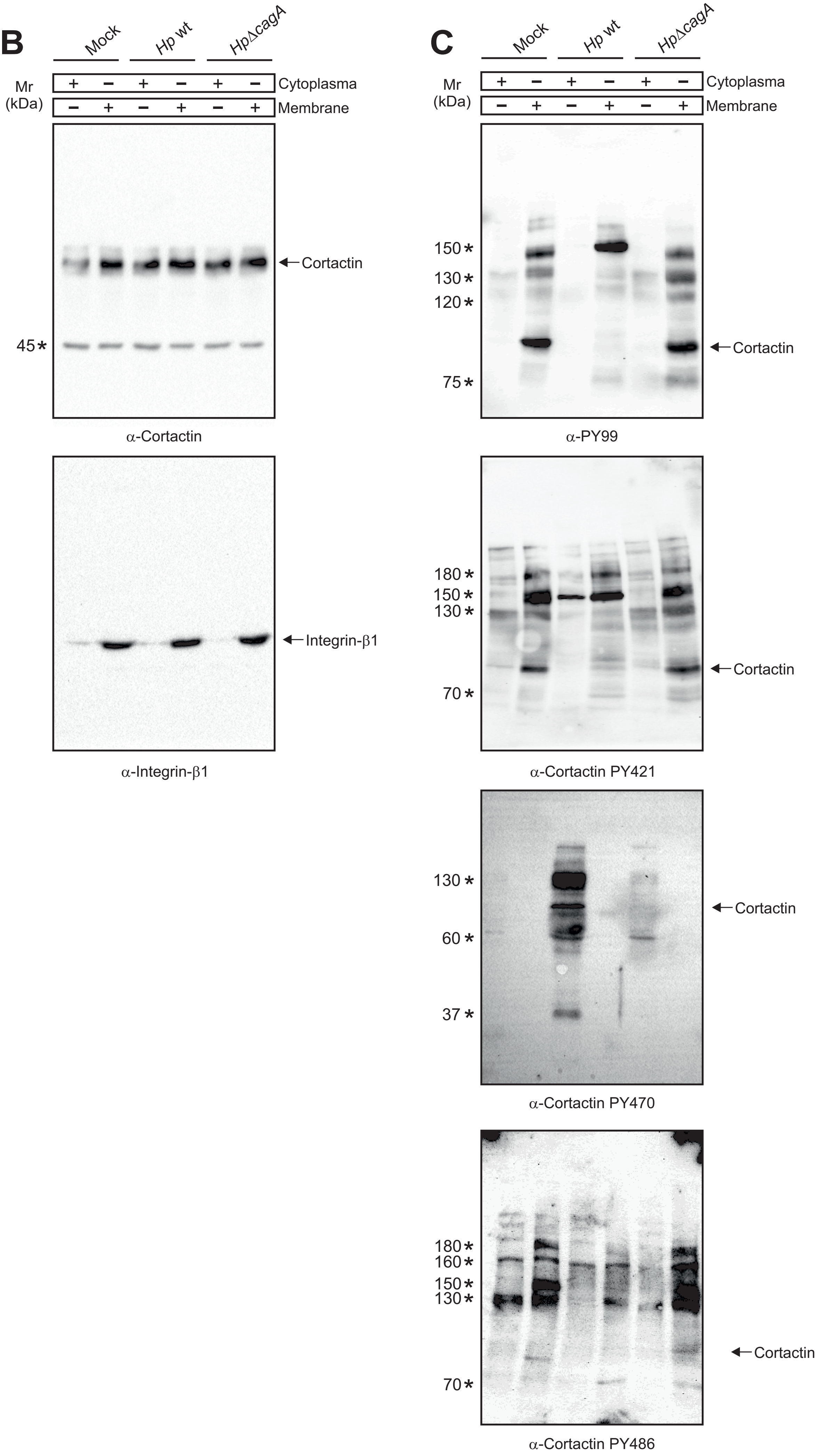
B
Mock
Hp wt
HpΔcagA
Mr
(kDa)
+ − + − + −
Cytoplasma
− + − + − +
Membrane
Cortactin
45 *
α-Cortactin
Integrin-β1
α-Integrin-β1
C
Mock
Hp wt
HpΔcagA
Mr
(kDa)
+ − + − + −
Cytoplasma
− + − + − +
Membrane
150 *
130 *
120 *
Cortactin
75 *
α-PY99
180 *
150 *
130 *
Cortactin
70 *
α-Cortactin PY421
130 *
Cortactin
60 *
37 *
α-Cortactin PY470
180 *
160 *
150 *
130 *
Cortactin
70 *
α-Cortactin PY486

Figure 2

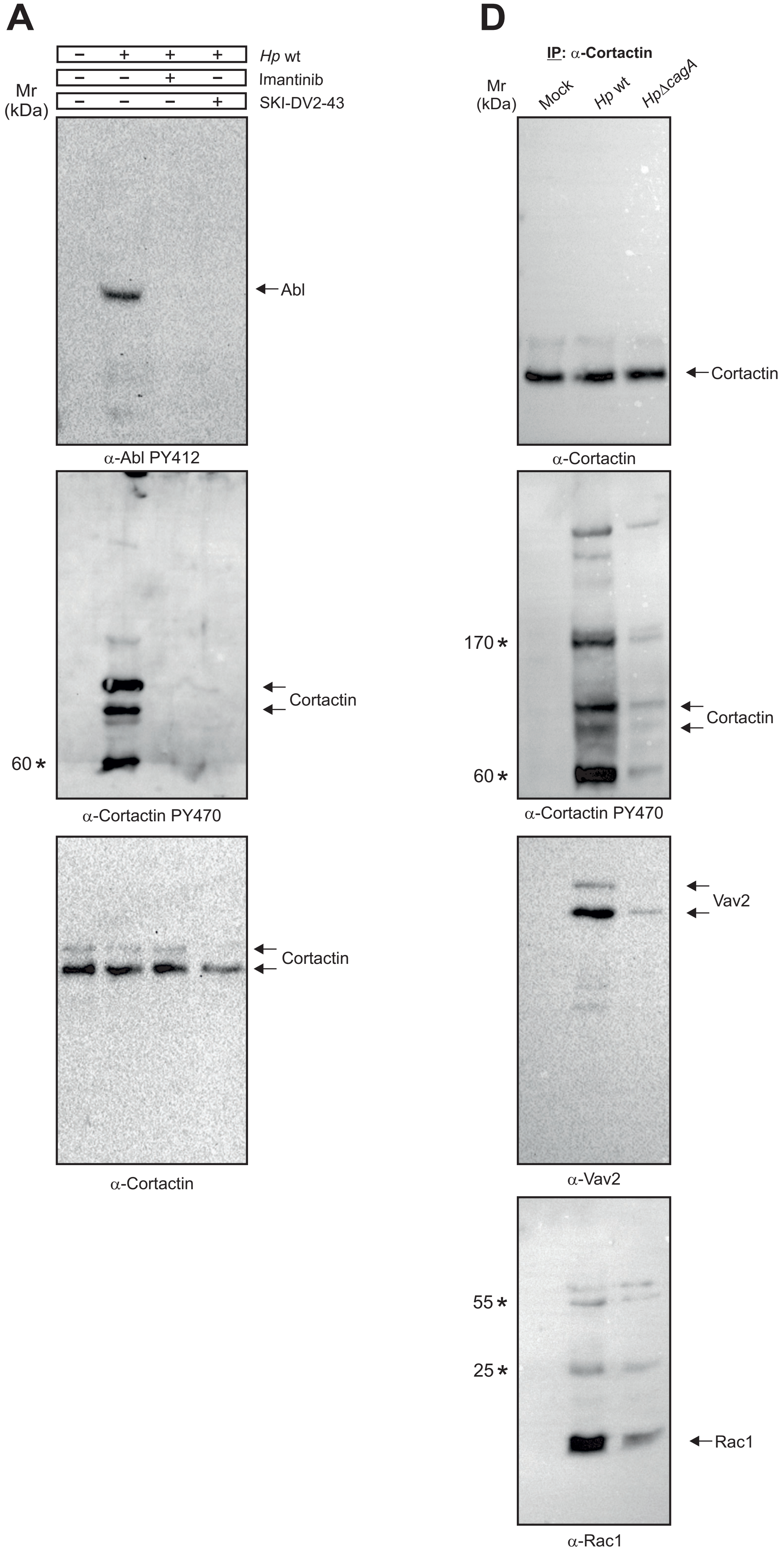
A
Hp wt
Imantinib
SKI-DV2-43
Mr
(kDa)
Abl
α-Abl PY412
60
Cortactin
α-Cortactin PY470
Cortactin
α-Cortactin
D
IP: α-Cortactin
Mr
(kDa)
Mock
Hp wt
HpΔcagA
Cortactin
α-Cortactin
170
60
Cortactin
α-Cortactin PY470
Vav2
α-Vav2
55
25
Rac1
α-Rac1

Figure 3

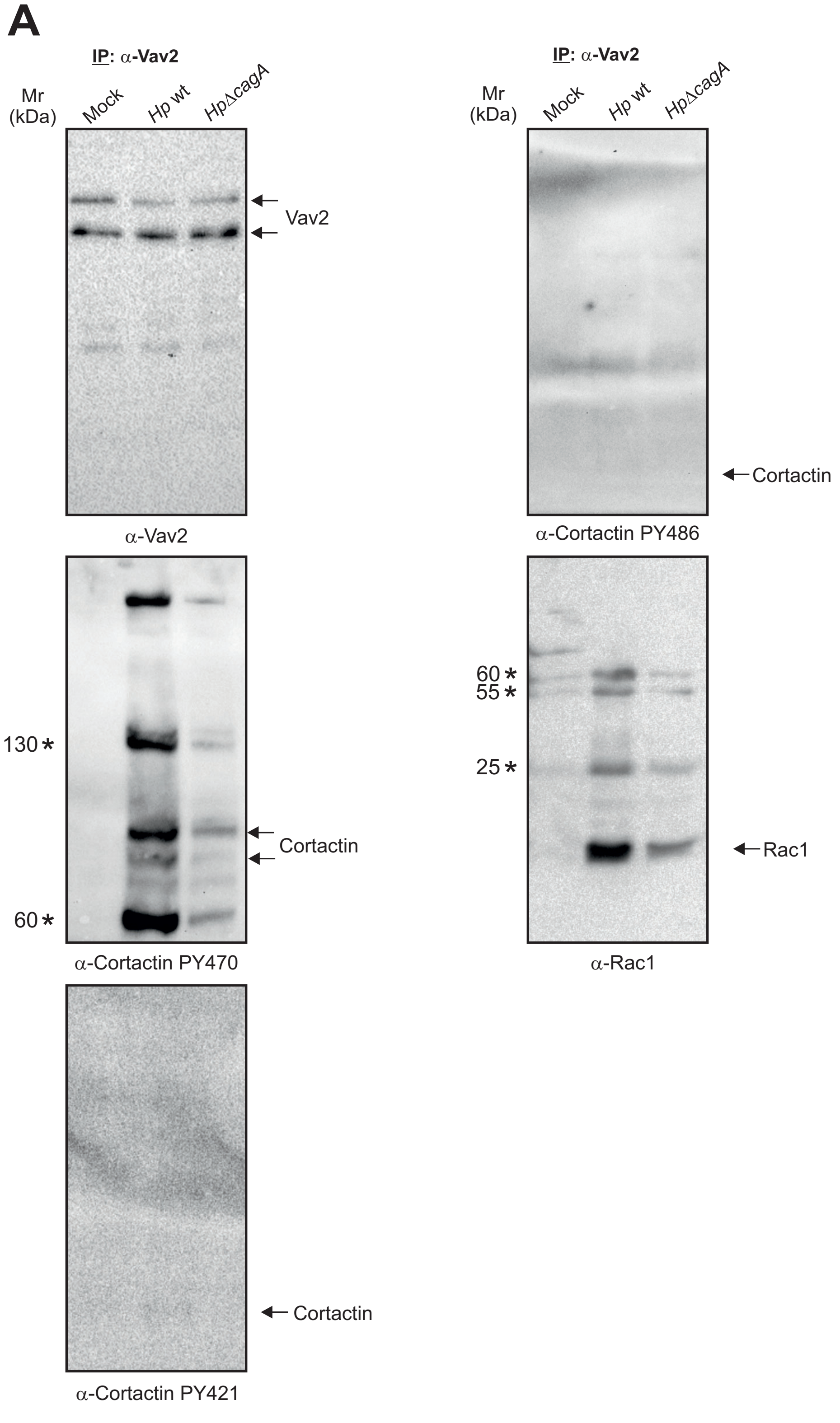
A
IP: α-Vav2
Mr (kDa)
Mock
Hp wt
HpΔcagA
Vav2
α-Vav2
130 *
60 *
Cortactin
α-Cortactin PY470
Cortactin
α-Cortactin PY421
IP: α-Vav2
Mr (kDa)
Mock
Hp wt
HpΔcagA
Cortactin
α-Cortactin PY486
60 *
55 *
25 *
Rac1
α-Rac1

Figure 4

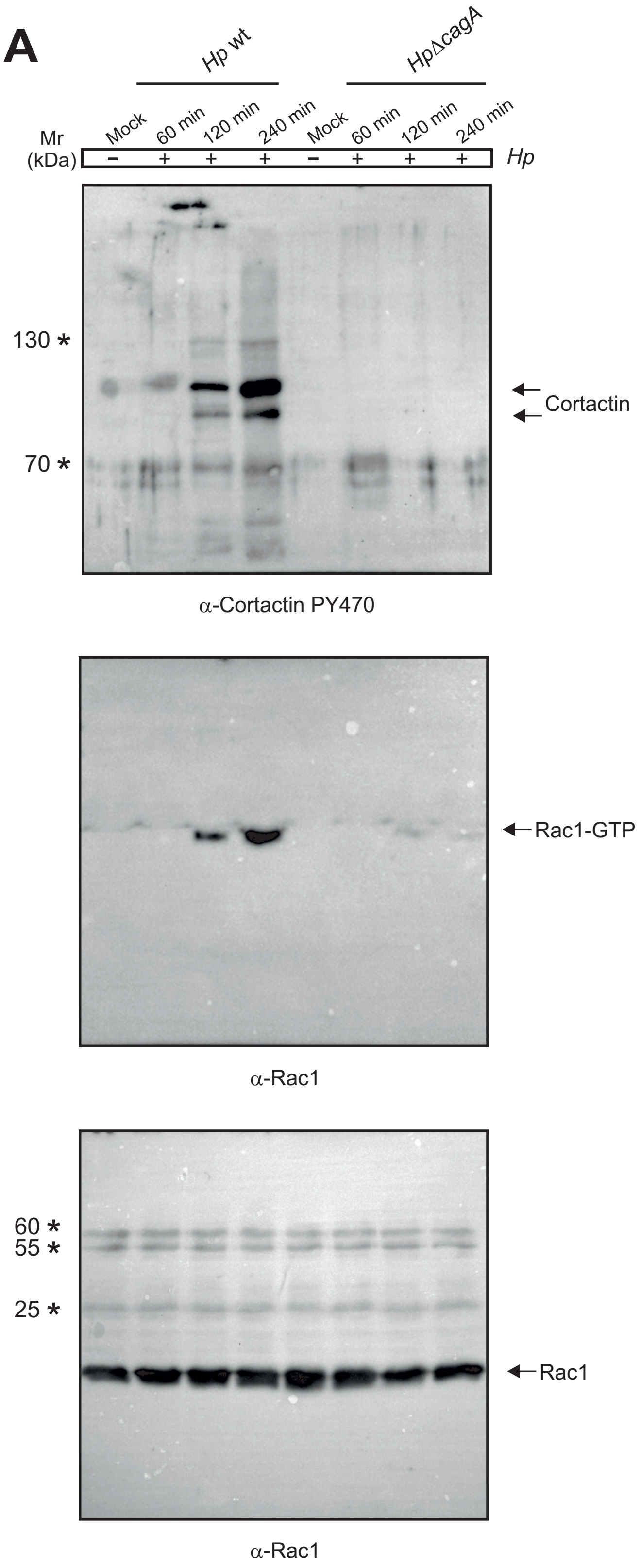
A
Hp wt
HpΔcagA
Mr
(kDa)
Mock
60 min
120 min
240 min
Mock
60 min
120 min
240 min
− + + + − + + +
Hp
130 *
70 *
Cortactin
α-Cortactin PY470
Rac1-GTP
α-Rac1
60 *
55 *
25 *
Rac1
α-Rac1

Figure 5

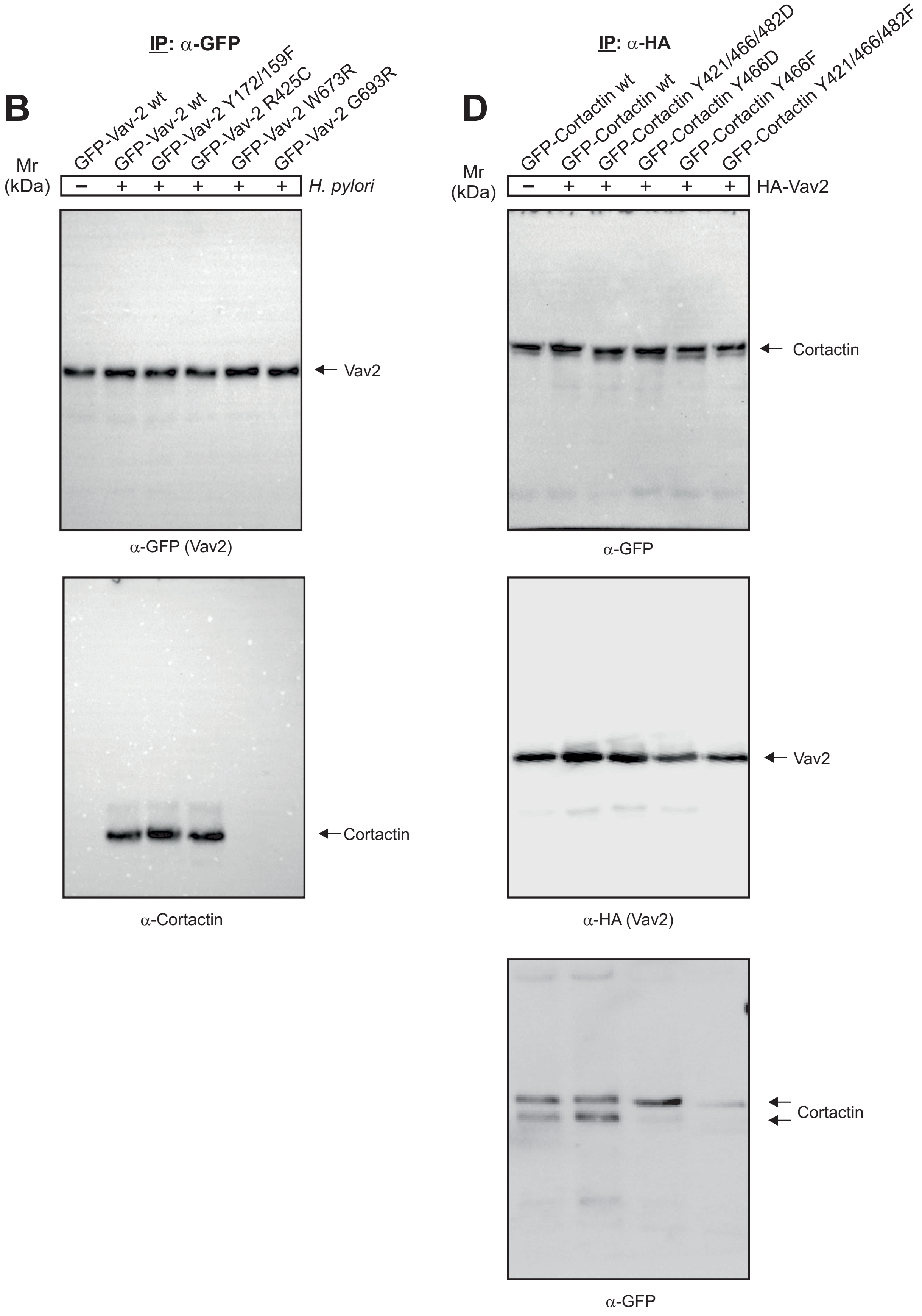

IP: α-GFP
B
GFP-Vav-2 wt
GFP-Vav-2 wt
GFP-Vav-2 Y172/159F
GFP-Vav-2 R425C
GFP-Vav-2 W673R
GFP-Vav-2 G693R
Mr
(kDa)
− + + + + +
H. pylori
Vav2
α-GFP (Vav2)
Cortactin
α-Cortactin
IP: α-HA
D
GFP-Cortactin wt
GFP-Cortactin wt
GFP-Cortactin Y421/466/482D
GFP-Cortactin Y466D
GFP-Cortactin Y466F
GFP-Cortactin Y421/466/482F
Mr
(kDa)
− + + + + +
HA-Vav2
Cortactin
α-GFP
Vav2
α-HA (Vav2)
Cortactin
α-GFP

Figure 6

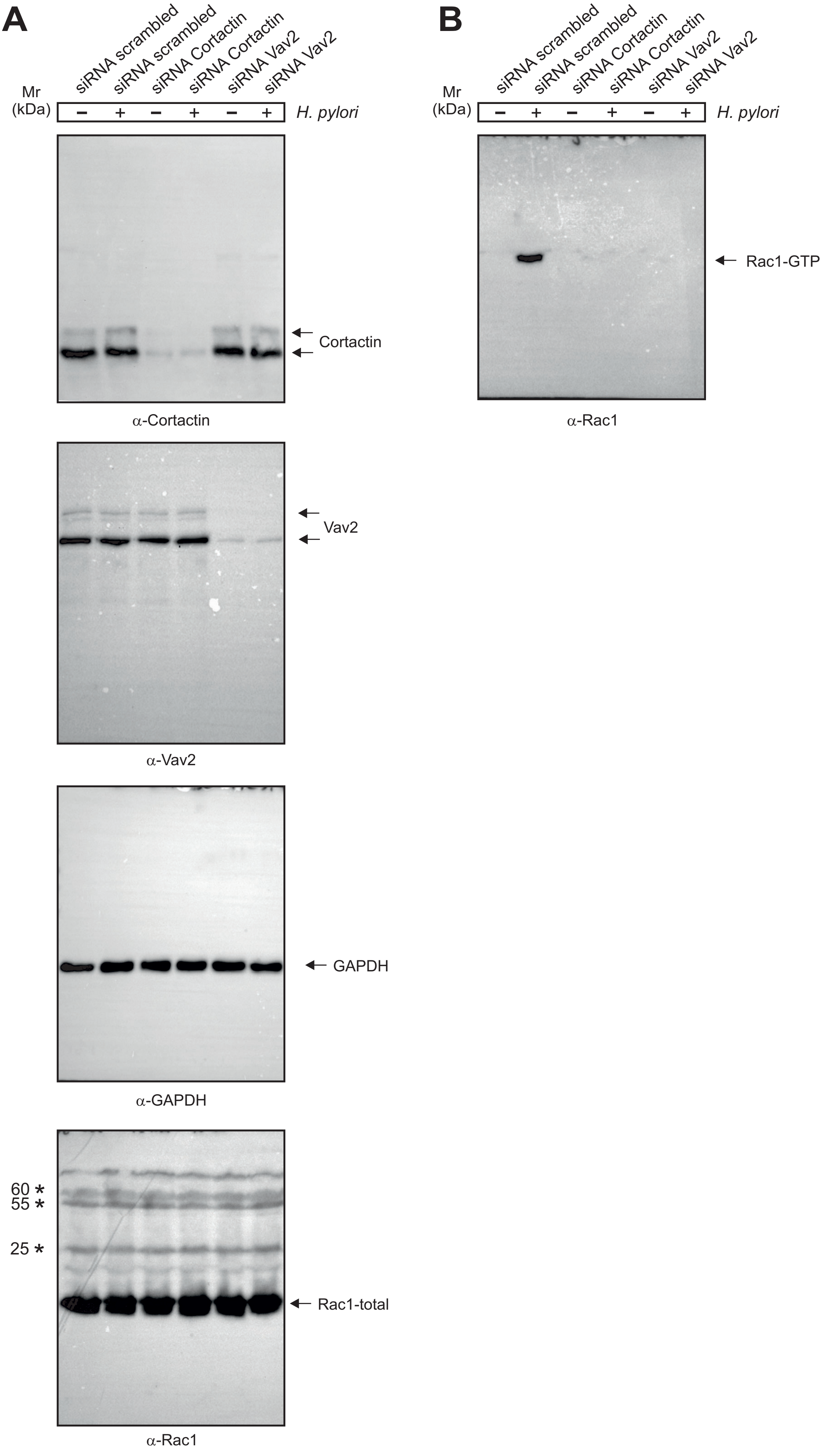

A
siRNA scrambled
siRNA scrambled
siRNA Cortactin
siRNA Cortactin
siRNA Vav2
siRNA Vav2
Mr
(kDa)
− + − + − +
H. pylori
Cortactin
α-Cortactin
Vav2
α-Vav2
GAPDH
α-GAPDH
60 *
55 *
25 *
Rac1-total
α-Rac1
B
siRNA scrambled
siRNA scrambled
siRNA Cortactin
siRNA Cortactin
siRNA Vav2
siRNA Vav2
Mr
(kDa)
− + − + − +
H. pylori
Rac1-GTP
α-Rac1
