# Supplementary material for: Helicobacter pylori CagA Induces Cortactin Y-470 Phosphorylation-Dependent Gastric Epithelial Cell Scattering via Abl, Vav2 and Rac1 Activation
Source: Cancers (Basel). 2021 Aug 23;13(16):4241. doi: 10.3390/cancers13164241 (PMC8391897; doi:10.3390/cancers13164241)
Supplement: Supplementary file 1 [file cancers-13-04241-s001.zip › Table S1.pdf]

cancers

MDPI

Suplementary

Helicobacter pylori CagA Induces Cortactin Y-470 Phosphorylation-Dependent Gastric Epithelial Cell Scattering via Abl, Vav2 and Rac1 Activation

Nicole Tegtmeyer, Aileen Harrer, Klemens Rottner and Steffen Backert

Table S1. Key resources of the present study.

Reagent	Source	Identifier
**Antibodies**		
Mouse monoclonal anti-GAPDH	Santa Cruz Biotechnology, Dallas, TX, USA	Cat# sc-47724; RRID:AB_627678
Mouse monoclonal anti-phosphotyrosine PY99	Santa Cruz Biotechnology, Dallas, TX, USA	Cat# sc-7020; RRID:AB_628123
Rabbit polyclonal anti-CagA	Austral Biologicals, San Ramon, CA, USA	Cat# HPP-5003-9; RRID: AB_10920428
Mouse monoclonal anti-β-Actin	Sigma-Aldrich, St. Louis, MO, USA	Cat# A5441, RRID:AB_476744
Mouse monoclonal anti-AIIB2 integrin-β1	Developmental Studies Hybridoma Bank, Iowa City, IA, USA	Cat#AB_528306; RRID:AB_528306
Rabbit polyclonal anti-Vav2	Santa Cruz Biotechnology, Dallas, TX, USA	Cat# sc-20803; RRID:AB_793922
Mouse monoclonal anti-Rac1	Cytoskeleton, Denver, CO, USA	Cat# ARC03; RRID:AB_2721173
Mouse monoclonal anti-Cortactin	Merck-Millipore, Burlington, MA, USA	Cat# 05-180; RRID:AB_309647
Rabbit polyclonal anti-Cortactin, phospho-specific (Tyr421)	Merck-Millipore, Burlington, MA, USA	Cat# AB3852; RRID:AB_177443
Rabbit polyclonal anti-Cortactin, phospho-specific (Tyr466)	Merck-Millipore, Burlington, MA, USA	Cat# AB3795; RRID:AB_569553
Rabbit polyclonal anti-Cortactin, phospho-specific (Tyr486) antibody	Merck-Millipore, Burlington, MA, USA	Cat# AB3853; RRID:AB_177444
Rabbit polyclonal anti-Abl, phospho-specific (Y412)	Sigma-Aldrich, St. Louis, MO, USA	Cat# 07-788; RRID:AB_612042
Rabbit polyclonal anti-GFP	Thermo Fisher Scientific, Waltham, MA, USA	Cat# A-11122; RRID:AB_221569
Goat polyclonal anti-Rabbit IgG (H + L) Cross-Adsorbed, FITC	Thermo Fisher Scientific, Waltham, MA, USA	Cat# F-2765, RRID:AB_253652
Rabbit polyclonal anti-Vav2	Proteintech, Rosemont, IL, USA	Cat# 21924-1-AP, RRID:AB_11182283
Goat anti-Rabbit IgG (H + L), HRP	Thermo Fisher Scientific, Waltham, MA, USA	Cat# 31462; RRID:AB_228338
Goat anti-Mouse IgG, IgM (H + L), HRP	Thermo Fisher Scientific, Waltham, MA, USA	Cat# 31446; RRID:AB_228318
**Bacterial Strains**		
Helicobacter pylori strain P1	[1]	not available

Helicobacter pylori strain P12	[2]	not available
**Inhibitors**		
Imatinib mesylate	Sigma-Aldrich, St. Louis, MO, USA	Cat# SML1027
SKI-DV2-43	[3]	not available
**Commercial Kits**		
Bradford assay kit	Bio-Rad, Hercules, CA, USA	Cat# 5000002
Rac1 activation assay kit	Cytoskeleton, Denver, CO, USA	Cat# BK035
**Cell Lines**		
AGS	ATCC, Manassas, VA, USA	Cat# CRL-1739; RRID:CVCL_0139
**siRNA Oligonucleotides**		
Cortactin siRNA (h)	Santa Cruz Biotechnology, Dallas, TX, USA	Cat# sc-35093
Control siRNA-A	Santa Cruz Biotechnology, Dallas, TX, USA	Cat# sc-37007
Vav2 siRNA (h)	Santa Cruz Biotechnology, Dallas, TX, USA	Cat# sc-41738
Rac1 siRNA (h)	Santa Cruz Biotechnology, Dallas, TX, USA	Cat# sc-36351
**Recombinant DNA Plasmids**		
Mouse Cortactin wt-EGFP	[4]	not available
Mouse Cortactin Y421/466/482F-EGFP	[4]	not available
Mouse Cortactin Y421/466/482D-EGFP	[4]	not available
Mouse Cortactin Y466F-EGFP	[4]	not available
Mouse Cortactin Y466D-EGFP	[4]	not available
Human Vav2 wt (HA-tagged)	S. Moores (Harvard Medical School, Boston, USA)	not available
Human Vav2 wt-EGFPC2	[5]	not available
Human Vav2 Y159F/Y172F-EGFPC2	[5]	not available
Human Vav2 R425C-EGFPC2	[5]	not available
Human Vav2 W673R-EGFPC2	[5]	not available
Human Vav2 G693R-EGFPC2	[5]	not available
**Software and Algorithms**		
GraphPad Prism v8.0	GraphPad Software, San Diego, CA, USA	www.graphpad.com (accessed on: 20 August 2021)
ImageJ-win64 v2.0	[6]	https://imagej.nih.gov/ij/ (accessed on: 20 August 2021)
LAS AF v4.0	Leica Microsystems, Wetzlar, Germany	https://www.leica-microsystems.com (accessed on: 20 August 2021)
Image Lab software v6.1	Bio-Rad, Hercules, CA, USA	https://www.bio-rad.com/de-de/product/image-lab-software?ID=KRE6P5E8Z (accessed on: 20 August 2021)

References

1. Backert, S.S.; Ziska, E.E.; Brinkmann, V.V.; Zimny-Arndt, U.U.; Fauconnier, A.; Jungblut, P.P.; Naumann, M.M.; Meyer, T.F. Translocation of the Helicobacter pylori CagA protein in gastric epithelial cells by a type IV secretion apparatus. *Cell. Microbiol.* **2000**, *2*, 155–164, doi:10.1046/j.1462-5822.2000.00043.x
2. Fischer, W.; Windhager, L.; Rohrer, S.; Zeiller, M.; Karnholz, A.; Hoffmann, R.; Zimmer, R.; Haas, R. Strain-specific genes of Helicobacter pylori: genome evolution driven by a novel type IV secretion system and genomic island transfer. *Nucleic Acids Res.* 2010, *38*, 6089–6101, doi:10.1093/nar/gkq378.
3. von Bubnoff, N.; Veach, D.R.; Miller, W.T.; Li, W.; Sänger, J.; Peschel, C.; Bornmann, W.G.; Clarkson, B.; Duyster, J. Inhibition of wild-type and mutant Bcr-Abl by pyrido-pyrimidine-type small molecule kinase inhibitors. *Cancer Res.* 2003, *63*, 6395–6404.
4. Tegtmeyer, N.; Wittelsberger, R.; Hartig, R.; Wessler, S.; Martinez-Quiles, N.; Backert, S. Serine phosphorylation of cortactin controls focal adhesion kinase activity and cell scattering induced by *Helicobacter pylori*. *Cell Host Microbe* 2011, *9*, 520–531.
5. Tamás, P.; Solti, Z.; Bauer, P.; Illés, A.; Sipeki, S.; Bauer, A.; Downward, J.; Buday, L. Mechanism of epidermal growth factor regulation of Vav2, a guanine nucleotide exchange factor for Rac. *J. Biol. Chem.* 2003, *278*, 5163–5171.
6. Schneider, C.A.; Rasband, W.S.; Eliceiri, K.W. NIH Image to ImageJ: 25 years of image analysis. *Nat. Methods* **2012**, *9*, 671–675, doi:10.1038/nmeth.2089.
